# Risk assessment of pre-hospital trauma airway management by anaesthesiologists using the predictive Bayesian approach

**DOI:** 10.1186/1757-7241-18-22

**Published:** 2010-04-21

**Authors:** Stephen JM Sollid, Hans Morten Lossius, Anders R Nakstad, Terje Aven, Eldar Søreide

**Affiliations:** 1 Department of Research and Development, Norwegian Air Ambulance Foundation, Drøbak, Norway; 2 Department of Anaesthesiology and Intensive Care, Stavanger University Hospital, Stavanger, Norway; 3 Department of Surgical Sciences, University of Bergen, Bergen, Norway; 4 Air Ambulance Department, Oslo University Hospital, Oslo, Norway; 5 Department of Industrial Economics, Risk Management and Planning, University of Stavanger, Stavanger, Norway

## Abstract

**Introduction:**

Endotracheal intubation (ETI) has been considered an essential part of pre-hospital advanced life support. Pre-hospital ETI, however, is a complex intervention also for airway specialist like anaesthesiologists working as pre-hospital emergency physicians. We therefore wanted to investigate the quality of pre-hospital airway management by anaesthesiologists in severely traumatised patients and identify possible areas for improvement.

**Method:**

We performed a risk assessment according to the predictive Bayesian approach, in a typical anaesthesiologist-manned Norwegian helicopter emergency medical service (HEMS). The main focus of the risk assessment was the event where a patient arrives in the emergency department without ETI despite a pre-hospital indication for it.

**Results:**

In the risk assessment, we assigned a high probability (29%) for the event assessed, that a patient arrives without ETI despite a pre-hospital indication. However, several uncertainty factors in the risk assessment were identified related to data quality, indications for use of ETI, patient outcome and need for special training of ETI providers.

**Conclusion:**

Our risk assessment indicated a high probability for trauma patients with an indication for pre-hospital ETI not receiving it in the studied HEMS. The uncertainty factors identified in the assessment should be further investigated to better understand the problem assessed and consequences for the patients. Better quality of pre-hospital airway management data could contribute to a reduction of these uncertainties.

## Introduction

Pre-hospital endotracheal intubation (ETI) has been considered the gold standard for airway protection and to ensure oxygenation and controlled ventilation in severely injured patients [1-3]. Despite this, studies on the clinical impact of pre-hospital ETI are divergent in their conclusions. Some studies indicate an increased survival related to pre-hospital ETI [4,5], whereas others indicate the opposite [6-8]. Several authors have claimed that pre-hospital ETI is associated with poor quality and high rates of complications that are more likely to kill than to save the patient [9-13]. Securing the airway by ETI represents a complex intervention [14] consisting of several critical factors and events. The poor quality and adverse events may be linked to choice of procedure (with or without drugs); lack of provider experience, training and exposure; or insecure and complicated treatment environments [15-17]. Despite high success rates with ETI [18], even airway experts like anaesthesiologists in emergency medical services (EMS) may face challenges when managing the airway of traumatised patients outside the hospital [19-21].

These challenges are closely related to quality of care and need to be addressed and investigated beyond counting complications and success rates.

Risk assessment methods are useful to investigate complex systems and provide insight into risks, but also to identify factors that influence risk to guide risk reducing measures and improve quality [22]. Many regard predictive risk assessments as especially well suited to health care issues because of their ability to include human factors in the assessment [22-24]. The novel predictive Bayesian approach in particular has been advocated for this use [22,25]. Still, the experience with the use of such risk assessment methods in health care is limited. The predictive Bayesian approach focuses on observable quantities: the actual population and the knowledge available [22,25]. Because this approach avoids the use of fictional parameters, it is regarded as a simple predictive risk assessment that is well suited for the study of health care issues [22,25].

The aim of this study was to investigate the quality of care in pre-hospital airway management by anaesthesiologists in patients with severe trauma and to identify possible areas for improvement. Specifically, we assessed the risk of a trauma patient not receiving pre-hospital ETI when there was an indication for ETI. The risk assessment was performed in a typical Norwegian HEMS.

## Methods

The risk assessment was performed according to the principles of the predictive Bayesian approach [22,25,26]. Initially, we defined the adverse event of interest as "Patient arriving in the emergency department (ED) with an unsecured airway when the indication to secure it was given pre-hospital", hereafter referred to as the "top event". The focus of the risk assessment was to determine the probability of the event and its consequences within the system analysed, to reflect on the uncertainties involved and to analyse the process leading to the event and its consequences.

### Fault tree

To analyse the causation leading to the event of interest, we constructed a fault tree. Fault trees are logical descriptions of the cumulative effects of faults within a system that show cause and effect relations among basic or initiating events that culminate in the adverse event of interest [24,27].

### Risk influencing factors

We then incorporated risk influencing factors into the risk description to include human and organisational factors in the assessment. The risk influencing factors were defined by three of the authors (SS, HML and AN), all experienced HEMS physicians. We also gave the risk influencing factors a score to reflect their quality and performance in the system assessed. To simplify, we only used three score levels: good, average and poor (Table 1). This simplification is in line with previous work [28].

**Table 1:** The definitions of the possible states of the risk influencing factors (RIF).

RIF	Possible states of RIFs		
	Poor	Average	Good
Culture and Attitudes	- Does not adhere to current recommendations or guidelines for advanced pre-hospital airway management. - Ignores good practice - Relies only on own opinion of what is best practice - Thinks that own skills are sufficient and that there is no need for practice outside clinical practice - Not aware of or neglects use of protocol - Overly confident in own ability to handle complications - Does not believe that serious complications will occur - Performs procedure for the benefit of the procedure, not to improve patient condition - Does not believe in protocols - No formal training of new providers - Performed by an unsupervised, inexperienced provider - Does not recognise experience and practise of other related services - Do not acquire new knowledge on a regular basis - No culture for feedback from receiving hospital	- Between good and poor	- Adheres to current recommendations and guidelines for advanced pre-hospital airway management, uses them in daily practice. - Has back-up from experienced provider - Positive attitude towards use of protocol to improve procedure safety - Prioritises patient safety - Formal training program for new providers - Takes preventive measures to avoid complications - Learns from own experience and complications ◦ Individual ◦ Department - Open learning environment - Novice operators under direct supervision from experienced operator - Interacts with other services to improve quality - Has "system" of acquiring feedback from receiving hospitals
			
Providers experience and knowledge	- Not competent in advanced airway management - Unfamiliar with difficult airway algorithm - Has no strategy for checking the patency of the airway after the procedure - Is not up to date on current recommendations and guidelines for advanced pre-hospital airway management - No defined relevant role model for own activity - Focus on own standing and career rather than patient outcome - Random assistant during airway procedures	- Competent in advanced airway management - Knows difficult airway algorithm - Checks patency of the airway at regular intervals - Has limited knowledge of current recommendations and guidelines for advanced pre-hospital airway management - Has trained assistant (that is integrated in the crew) for airway procedures, but makes irregular use of assistance	- Competent in advanced airway management - Competent in difficult airway management - Familiar with local back-up airway equipment - Familiar with potential airway complications in the pre-hospital setting and the handling of these - Monitors the patency of the airway after the procedure - Knows the current recommendations and guidelines for advanced pre-hospital airway management, especially the use of tracheal intubation. - Uses trained assistant (that is integrated in the crew) during airway procedures
			
System	- System has no policy on hiring providers experienced in pre-hospital medicine - Most providers are inexperienced in pre-hospital medicine or are in-training - There is no system for training or retraining the providers in advanced airway management - No system for quality assurance - No formal R&D activities - Techniques and equipment used for advanced airway management is not up to date with current standards	- System hires mostly providers experienced in pre-hospital medicine or specialists within their field - The providers are trained in some of the skills and procedures related to advanced airway management at regular interval or all procedures at irregular intervals	- System hires only providers experienced in pre-hospital medicine and specialists within their field - All providers are trained and retrained regularly in all skills and procedures related to advanced airway management, including rescue techniques - Techniques and equipment used in advanced airway management are up to date with current standards - Service registers activity data from advanced airway management and uses data for quality improvement and research
			
Protocol compliance	- No protocol available or available protocol is not followed - Protocol do not match provider competence	- Protocol available, but does not give a clear framework for the procedure (see "good") - Partially follows protocol	- Protocol for advanced pre-hospital airway management exists. - Protocol defines framework for the procedure - Protocol defines measures to improve quality and safety of procedure - Protocol defines team roles - Protocol is followed in all cases - Protocol is regularly updated to comply with current knowledge

To calculate the probability of the top event in the analysed system based on the risk influencing factors, we first agreed on a set of probabilities for the basic events in a generic system. We assigned probabilities for three cases: all risk influencing factors poor, all risk influencing factors average and all risk influencing factors good. Based on these values, we then calculated the appropriate "adjustment factor" needed in the analysis when the status and weighted influence of the risk influencing factors were taken into consideration. For this calculation, we used the approach described by Aven [28], adjusted for our purposes with only three score levels.

The risk influencing factors were then assigned their appropriate scores within the system assessed by our expert judgement, and the probability of the initiating event was computed, given the assigned quality and performance of the risk influencing factors.

The knowledge basis used for the assessment is found in Table 2.

**Table 2:** Knowledge basis for the risk assessment

i)	A review of the literature on outcomes of pre-hospital advanced airway management (unpublished), including a recent Cochrane review on the same topic [30].
ii)	A recent survey of Norwegian helicopter emergency medical service (HEMS) physicians' own perceptions of the safety and quality of pre-hospital airway management in their system [19].
iii)	Audit of data from patients with severe traumatic injury treated by the assessed HEMS systems in the 1994 -- 2005 period (unpublished data, manuscript in preparation).
iv)	Expert judgement by three of the authors (SS, HML and ARN) as experienced HEMS physicians and one independent HEMS physician from the system assessed.
v)	The literature cited in this study.

### Consequence analysis

To analyse the consequences of the event, we constructed an event tree. Event trees are logical structures that map out the possible consequences of the event of interest and the pathways leading up to the consequences [24]. It is important to notice that event trees are based on the assessors' interpretation of the causations leading up to the possible outcomes. This implies that the assessor has to make certain assumptions that need to be declared when the event tree is presented. Two important assumptions were made in our case. Firstly, that time is an important factor, meaning that delay in treatment impacts outcome. This was however not visualized in the fault tree. Secondly, the clinical state of the patient following the traumatic injury was not taken into consideration when estimating the outcomes, as this would make the assessment to complex.

The possible consequences of the initiating event were described based on the knowledge basis, our knowledge of the system receiving the patients in the ED and the risk analysis and are presented in a risk matrix, where we defined four main categories with five different frequencies/probability categories.

### Uncertainty assessment and sensitivity analysis

In the uncertainty assessment, we identified uncertainty factors that were judged to have an influence on risk. Uncertainty factors are related to the interpretation of phenomena or assumptions made in the risk assessment (based on the knowledge available) that can turn out to be wrong in the future when better knowledge of the phenomena is available [29]. Their importance with respect to the effect on risk was categorised as minor, moderate or major based on the description by Flage and Aven [29]. This categorisation was case-specific for the system assessed and was based on the judgement of the assessor. To further investigate the importance of these uncertainty factors, we performed a sensitivity analysis where we investigated how changing the assumptions or interpretations made in the risk analysis would influence the result of the risk analysis. If changing them was found to be important for the risk indices (probabilities of adverse events and consequences) under consideration, it was assigned a high sensitivity; conversely, it was assigned a low sensitivity if changes had little or no influence on the risk indices [29]. Uncertainty factors subject to large uncertainties that would also change the risk indices by even a small amount were regarded as having a significant effect on risk. These uncertainty factors should be subjected to further investigation to increase the knowledge of the phenomena.

## Results

**Figure 1:**
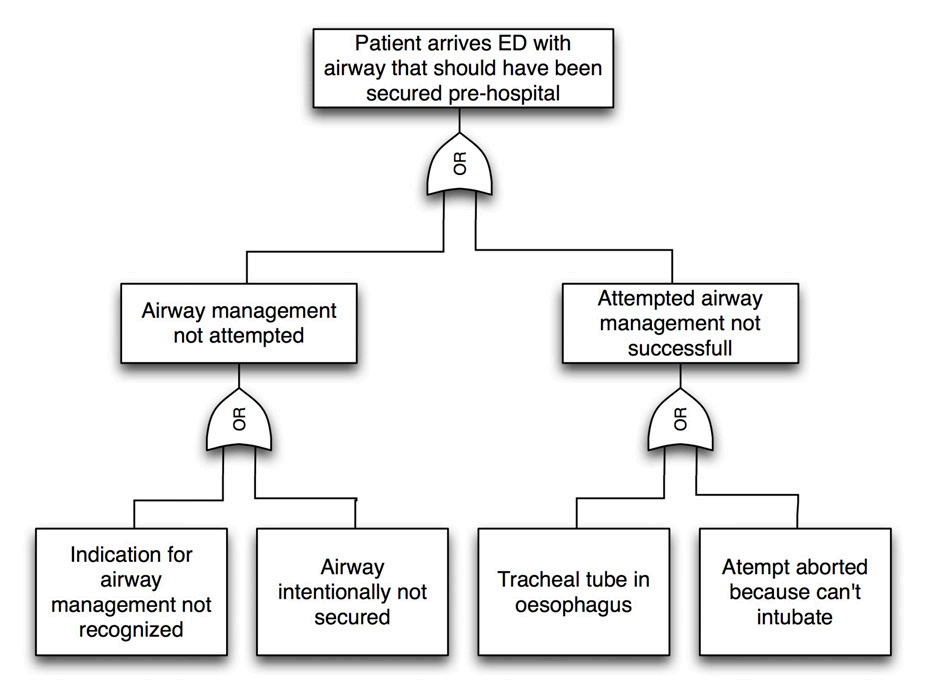
**Fault tree visualising the process leading up to the top event**.

**Figure 2:**
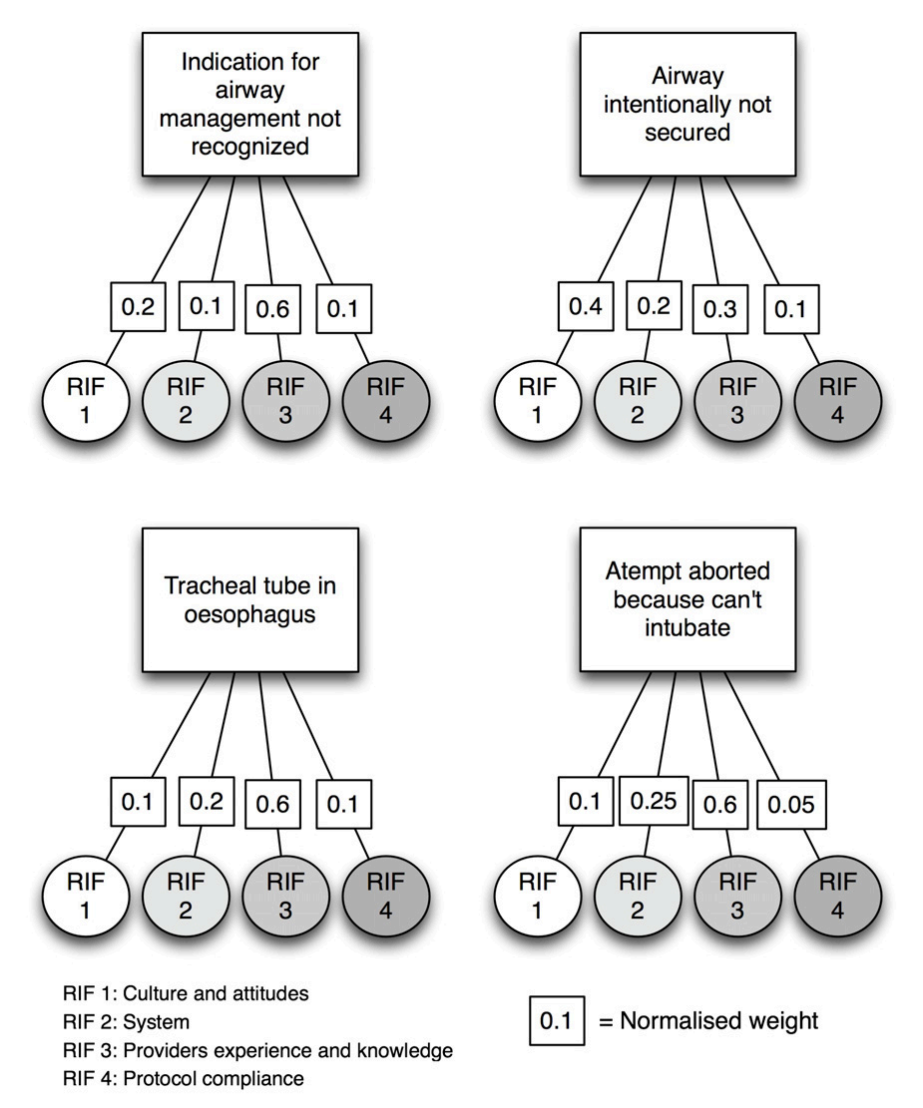
**Influence diagram illustrating the impact of the risk influencing factors on the basic events of the fault tree**. The impact is shown as normalized weights, meaning that the sum of impacts on each basic event is 1.0 and the impact of each risk influencing factor represents a fraction of this.

In this study we identified four basic events leading up to the main event, thus constructing a fault tree as shown in Figure 1. Based on our evaluation, we decided that all risk influencing factors influenced all basic events, but that the weighted influence was different for each basic event. Figure 2 shows a simple influence diagram demonstrating the influence of the risk influencing factors on the basic events. Based on our knowledge and judgement of the system analysed we assigned the following scores for the risk influencing factors:

- Culture and attitudes: average

- System: poor

- Providers' experience and knowledge: average

- Protocol compliance: poor

**Figure 3:**
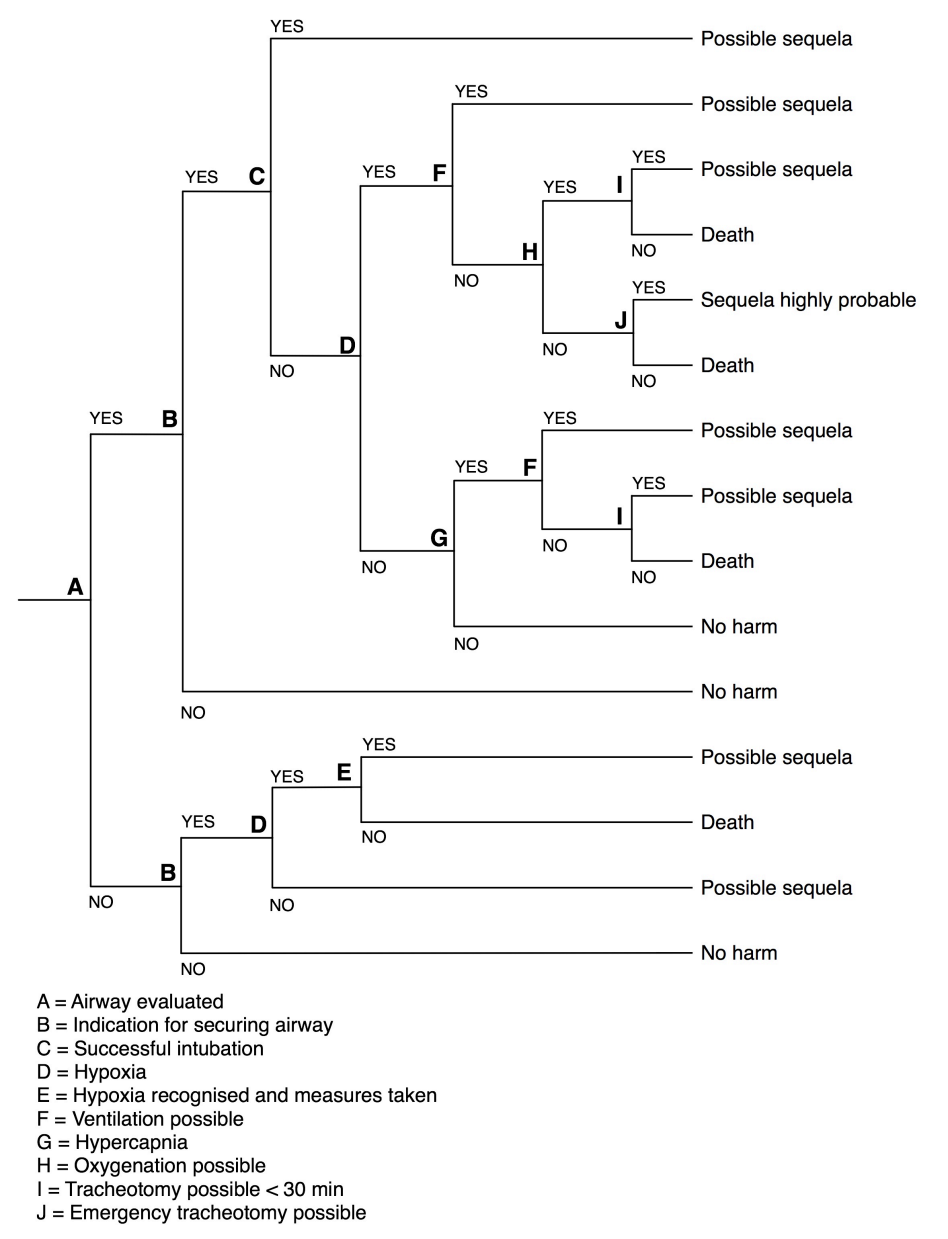
**Event tree visualising the possible outcomes of the event being assessed**.

**Figure 4:**
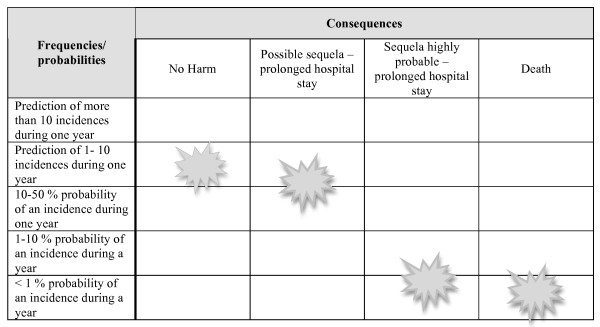
**Risk matrix with possible consequences of the event that a patient arrives in the emergency department without endotracheal intubation, when there was an indication for endotracheal intubation pre-hospital**. The figures indicate the assigned probabilities for the outcomes.

With the assigned risk influencing factor basis, we calculated the probability of the top event at 29%, meaning that we would expect the event to take place in 29 of 100 cases if we were to observe the system today. The event tree constructed for the consequence analysis is presented in Figure 3. In the consequence analysis we found the probability of "no harm" and "possible sequela with prolonged hospital stay" to be almost equal (Figure 4) given the event where the patient arrives in the ED without a secured airway when the indication for ETI was given pre-hospital. The consequence "possible sequelae with a prolonged hospital stay" was assigned a probability of at least 10-50% during one year, but less than 1-10 incidents occurring during one year (Figure 4). The probability of death following the event was assigned as less than 1% during one year (Figure 4).

Uncertainty factors identified in the uncertainty assessment are listed in Table 3. In the sensitivity analysis, we found none of the uncertainty factors to have significant sensitivity, but all factors regarding the system and culture and attitudes were assigned a potentially moderate significance, meaning that changing the factors would somewhat change the risk indices too.

**Table 3:** Uncertainty factors identified in the uncertainty assessment

		Effect on risk	
Uncertainty factor	Minor	Moderate	Major
Amount of training needed to maintain airway skills		x	
Need for special training in pre-hospital airway management		x	
Impact of patient's condition on consequences			x
Reliability of data recorded in patient charts			x
Criteria used to decide whether or not patient should be intubated		x	

## Discussion

In our risk assessment of the typical Norwegian anaesthesiologist-manned HEMS, we found that the probability of a patient arriving in the hospital without ETI when there was a pre-hospital indication for it was as high as 29%.

Most studies on pre-hospital advanced airway management report on complication- or success rates and are either retrospective [30,31] or prospective observational studies [12,32-35]. In both cases they only present the incidence of complications or successful ETI in the system where the data was collected at the time of collection. The transferability of the results to other systems or even to the same system may be questionable as other factors influence practice in other systems, and systems may change. Predictive risk assessments like the predictive Bayesian approach may provide a more correct picture of the problem today because it uses a broader knowledge basis (qualitative and quantitative data, and expert judgement) to assess the problem - or rather the risk - as it is today [22,25,26]. It is also tailored for the system being assessed in that it uses system specific or -relevant knowledge in the risk assessment [25]. This means that the risk indices assigned in the risk assessment of our top event expresses what we expect the risk to be today in the system assessed.

The predictive Bayesian approach is not only useful for describing a risk picture, it also provides us with a tool to visualise and analyse the causes leading up to the event of interest and to identify where improvements are needed and would have the greatest effect. From a patient safety perspective, this is essential: we often know there is a problem, but we do not have a sufficient understanding of the mechanisms causing the problem. Besides the initiating events in the fault tree, the risk influencing factors provide a good indication of where measures to influence the risk have impact. By changing the values of the assigned probabilities for the basic events in the fault tree, we can for example get an impression of where risk-reducing measures have the greatest impact. If all risk influencing factors in our case were scored as good, the probability of the top event could be reduced to 3%, or with all risk influencing factors scored as average, to 17%. Changing the system and culture and attitudes risk influencing factors to a level where they would be scored as good would change the assigned probability of the top event to 14%, whereas optimising only the providers' competence risk influencing factor to the level where it would be scored as good would lead to an assigned probability of 24%. This implies that risk-reducing measures in our system should focus on system and culture and attitudes.

To establish which aspects of these risk influencing factors that need improvement, we think a key task would be to establish why the physician in charge decides to refrain from securing the airway pre-hospital. In our audit of the HEMS studied (unpublished data, manuscript in preparation), which was part of the knowledge basis for the risk assessment, some physicians have commented that they abstained from pre-hospital ETI because of a short transport distance. The same data show that transport time was lower in the group that was intubated in the ED compared to those intubated pre-hospital. Further, the audit data indicate that the patients intubated pre-hospital were more severely injured than those intubated in the ED because of lower RTSs and GCSs. This might explaining why ETI was postponed in some cases: short transport distance and less serious injury. Still, the patients were intubated immediately upon arrival in the ED, indicating that there was a need to secure the airway. This raises the question of whether the postponed ETI had any impact on the patients' condition. A recent study from the Netherlands [36] showed a failure to adhere to guidelines for pre-hospital ETI in traumatic brain injury in almost half of the studied population. Further, the authors found a negative influence on respiratory and metabolic parameters in patients not intubated [36]. Other studies have also shown that poor oxygenation and ventilation may worsen outcome [37-39]. If we assert that hypoxia and hypoventilation is more likely to occur in a non-intubated patient, then to abstain from ETI to reduce scene to door time has the potential to harm the patient. The findings of a recent study also indicate that delayed treatment of critically injured patients until arrival in the trauma centre worsens outcome [40]. The potential effect of delayed treatment, in our case ETI, was regarded as important in the consequence analysis of our top event and was one of the reasons why we assigned a high probability of possible sequela and delayed hospital stay following the top event. Because we did not take the patients condition following the injury into account in the consequence analysis, we assigned the impact of the patient's condition on consequences to be an uncertainty factor with major effect on risk.

As the uncertainty analysis revealed there are other important uncertainty factors that influence the analysis at this point. Because we have little knowledge about why the physicians decided not to intubate in some cases the criteria used for deciding to intubate or not was assigned as an uncertainty factor. The quality of the audit data used in the assessment was also difficult to assess. For the purpose of the risk assessment we assumed that the quality of the data recorded in the patient charts would be good, but observed that the completeness of the records was variable. The reliability of the data was therefore assigned to have major effect on risk. Reliable and better quality data would obviously reduce both these uncertainties. For this purpose initiatives like the recent Utstein style template for registering data following pre-hospital airway management are important [41].

This risk assessment was performed for the Stavanger HEMS based on our knowledge of and experience with the system. The probabilities were assigned by the assessor as an expression of the assessor's uncertainty about the occurrence of the top event [22,25,26]. These probabilities are not estimates or predictions of some true value that is thought to exist, as they are often presented in classical risk assessment [22,25,26]. As such, they are subjective and conditional on the knowledge available about the system being assessed and the assessor's interpretation of this knowledge. Compared to traditional medical science, a risk assessment conducted according to the predictive Bayesian approach might seem like an exercise in subjective interpretation of data that is prone to assessor bias. Admittedly, this kind of risk assessment is heavily based on the assessors' own judgement, but the assessment process is methodical and traceable. The risk assessment does not attempt to present "truths" or "evidence", but rather focuses on what we can interpret from the knowledge available and what limitations, or rather what uncertainties, there are in the assessment. It is therefore important to declare and assess the uncertainty factors in the analysis. Triangulation is a term used in land surveying to determine the position of an object; by drawing a line to the object from at least three different observation points, the object's exact position can be determined. The same term is used to describe the method of analysing a problem using different sources of information and data and from different viewpoints in qualitative research to develop an overall interpretation [42] and is also, in our opinion, a valid analogy for assessments like the predictive Bayesian approach. In our case the knowledge basis illustrates the points of triangulation. We believe this to be the most important contribution of the predictive Bayesian approach to risk management in general and in particular to risk management in health care.

Although the risk assessment was limited to one specific Norwegian HEMS, we think the concerns raised may apply to other similar systems. Providing the best quality of care to seriously injured patients can be challenging and as our risk assessment indicates, the decision on when to perform ETI or not may be influenced by many factors. The predictive Bayesian approach provides a tool to perform the same risk assessment in other HEMS systems, provided the appropriate background knowledge from the specific system studied is used.

## Conclusion

Our risk assessment of a typical Norwegian HEMS indicated a high probability of the event in which a severely traumatised patient arrives in the hospital without ETI despite a pre-hospital indication for it. The consequence analysis also indicates a high probability of sequela for the patient following this event. There are, however, important uncertainty factors related to this assessment that need to be further investigated to improve our understanding of the event studied. We think better quality of pre-hospital airway management data would contribute to reduce these uncertainties.

## Competing interests

The authors declare that they have no competing interests.

## Authors' contributions

SS designed the study, collected the data, performed the statistical analysis and the risk analysis and drafted the manuscript. HL helped design the study, contributed to the statistical analysis and the risk analysis and helped draft the manuscript. AN contributed to the risk analysis and helped review the manuscript. TA contributed to the risk analysis and helped review the manuscript. ES helped design the study and collect the data and reviewed the manuscript. All authors read and approved the final manuscript.
